# Therapeutic Effects of Anti-Inflammatory and Anti-Oxidant Nutritional Supplementation in Retinal Ischemic Diseases

**DOI:** 10.3390/ijms25105503

**Published:** 2024-05-18

**Authors:** Deokho Lee, Zhongjie Fu, Ann Hellstrom, Lois E. H. Smith

**Affiliations:** 1 Department of Ophthalmology, Boston Children’s Hospital, Harvard Medical School, Boston, MA 02115, USA; 2 The Sahlgrenska Centre for Pediatric Ophthalmology Research, Department of Clinical Neuroscience, Institute of Neuroscience and Physiology, Sahlgrenska Academy, University of Gothenburg, 416 85 Gothenburg, Sweden

**Keywords:** nutrients, retinal ischemic diseases, anti-inflammation, neuroprotection, anti-neovascularization

## Abstract

Appropriate nutrients are essential for cellular function. Dietary components can alter the risk of systemic metabolic diseases, including cardiovascular diseases, cancer, diabetes, and obesity, and can also affect retinal diseases, including age-related macular degeneration, diabetic retinopathy, and glaucoma. Dietary nutrients have been assessed for the prevention or treatment of retinal ischemic diseases and the diseases of aging. In this article, we review clinical and experimental evidence concerning the potential of some nutritional supplements to prevent or treat retinal ischemic diseases and provide further insights into the therapeutic effects of nutritional supplementation on retinopathies. We will review the roles of nutrients in preventing or protecting against retinal ischemic diseases.

## 1. Introduction

**Figure 1:**
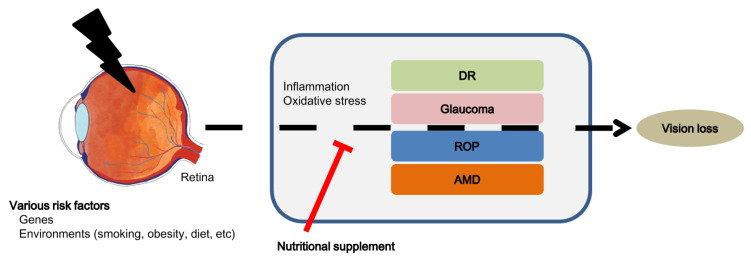
Schematic illustration of therapeutic effects of nutritional supplements in retinal ischemic diseases. Risk factors, including genes and environment (smoking, obesity, and diet), may cause retinal ischemic diseases (DR: diabetic retinopathy, glaucoma, ROP: retinopathy of prematurity, and AMD: age-related macular degeneration) via complex molecular pathways (including inflammation and oxidative stress), leading to vision loss. Nutritional supplementation (omega-3 polyunsaturated fatty acids, lutein + zeaxanthin, resveratrol, vitamin B3 and nicotinamide mononucleotide, vitamin B6, vitamin C, lactoferrin, crocetin, taurine, and palmitoylethanolamide) may prevent or protect against retinal ischemic diseases.

Nutrients can affect tissue health. Disease risk factors for obesity and metabolic diseases, cardiovascular diseases, and cancer include physical inactivity, smoking, and dietary habits [1]. Metabolic dysregulations can directly or indirectly affect the retina, leading to retinal diseases, including age-related macular degeneration (AMD), retinopathy of prematurity (ROP), diabetic retinopathy (DR), or glaucoma [2,3]. Inflammation and oxidative stress contribute to disease progression. Nutritional supplementation could positively affect oxidative stress and inflammation against pathology. In this article, we review recent nutritional supplementation as potential therapeutic strategies to prevent or treat retinal ischemic diseases (Figure 1) based on our own recent reports as well as a search of the related literature.

## 2. Nutrients in Retinal Diseases

### 2.1. Omega-3 Polyunsaturated Fatty Acids

Omega-3 polyunsaturated fatty acids (ω3 LCPUFAs) are found at high levels in fatty fish, such as salmon, mackerel, sardines, and anchovies [4]. Other dietary sources are nuts and seeds, such as flaxseed, chia seeds, walnuts, and plant oils: flaxseed oil, soybean oil, and canola oil. The dietary ingestion of fish containing ω3 LCPUFAs eicosapentaenoic acid (EPA), and docosahexaenoic acid (DHA) has a beneficial effect on many retinal diseases [5,6,7,8,9,10,11,12,13]. However, the dietary supplementation of DHA (350 mg/day) and EPA (650 mg/day) in a well-nourished patient cohort did not reduce the risk of advanced AMD in the Age-Related Eye Disease Study 2 (AREDS2) [14]. The reasons for the inconsistent observations of DHA supplementation in preventing retinopathies are unclear. It has been noted that DHA and EPA (ω3 LCPUFA) supplements may lower circulating arachidonic acid (AA) levels [15,16] and decrease the AA/DHA ratio in preterm infants [9]. We recently reported that low postnatal AA serum levels are strongly associated with clinical ROP development [17], suggesting that maintaining adequate AA levels might be required for DHA to protect against ROP. The oral supplementation of DHA and AA at 1:2 ratio improves visual acuity at 12 months of age in preterm infants, but a higher DHA/AA ratio does not show any additional improvement [18]. More recently, we have shown that oral DHA and AA at 1:2 ratio increases serum levels of both AA and DHA, and reduces severe ROP by 50% in very preterm infants [19,20].

Supplementation with ω3 LCPUFAs may reduce the onset and progression of neovascular retinal diseases [19,21,22,23]. We found that dietary ω3 LCPUFA, DHA, and EPA versus ω6 LCPUFA (AA) suppresses laser-induced choroidal neovascularization (CNV), modeling the inflammatory aspects of wet AMD, and suppresses hypoxia-induced retinal neovascularization in oxygen-induced retinopathy (modeling neovascularization in ROP and DR). These lipids also improve hyperglycemia-induced retinal vessel growth delay in rodents [22,24,25]. Adiponectin (APN), an adipokine mainly produced in white adipose tissue, reduces retinal inflammation and mediates the therapeutic effect of ω3 LCPUFAs [22,24,25]. We further found that cytochrome P450 oxidase 2C and 2J inhibition augments the inhibitory effects of ω3 LCPUFA on CNV formation [26,27]. Wang et al. recently showed that dietary ω3 PUFA diminishes hypoxia-induced retinal neovascularization by controlling microglial pyroptosis [28], an important mediator of neuro-inflammation [29].

Dietary ω3 LCPUFAs may also reduce diabetic complications. Based on data from a combined population of 1356 subjects with type 2 diabetes in the Multi-Ethnic Study of Atherosclerosis (MESA) and Genetics of Latino Diabetic Retinopathy (GOLDR) cohorts, the levels of DHA (ω3 LCPUFA) are inversely associated with the presence and severity of DR, suggesting that the increased consumption of the dietary sources of DHA might protect against retinal damage in type 2 diabetes [30]. Dietary ω3 LCPUFAs (DHA and EPA) versus ω6 LCPUFA (AA) suppresses retinal dysfunction in mice modeling type 2 diabetes mellitus [31]. However, no significant impact on retinal inflammatory responses is found in ω3 versus ω6 LCPUFA-fed diabetic mice [31], suggesting that other mechanistic pathways might be involved. We recently showed that in mice with neonatal hyperglycemia, ω3 and ω6 LCPUFAs influence retinal metabolism and retinal growth [22]. Further investigations are needed to more fully elucidate the role of ω3 LCPUFA in retinal health.

Blood DHA levels are lower in patients with primary angle-closure glaucoma versus people without glaucoma [32], and the low consumption of fatty fish is suggested to correlate with high-tension primary open-angle glaucoma [33]. Georgiou et al. found in a rat model of anterior ischemic optic neuropathy that ω3 LCPUFAs protect against retinal ganglion cell loss by reducing macrophage recruitment and promoting anti-inflammatory responses [34]. In a mouse model of autosomal dominant optic atrophy, Kalogerou et al. also showed that ω3 LCPUFA is neuroprotective possibly by modulating microglial and astrocytic activation, as well as through the suppression of apoptosis [35]. Moreover, oral supplementation with ω3 LCPUFAs reduces intraocular pressure in normotensive subjects [36], suggesting the therapeutic potential of ω3 LCPUFA to prevent the loss of ganglion cells secondary to ocular hypertension. Nguyen et al. suggested that dietary ω3 LCPUFA consumption reduces aging-induced increases in intraocular pressure in rats [37]. This effect was related to the modulation of aqueous outflow. Based on the accumulating evidence, ω3 LCPUFAs are a promising nutrient supplement for retinal diseases. However, the composition of supplemented ω3 and ω6 LCPUFAs and the underlying mechanisms need to be further defined.

### 2.2. Lutein + Zeaxanthin

The carotenoids lutein and zeaxanthin are found in the macula of the human retina [38,39] and are abundant in egg yolk and dark green leafy vegetables [40]. A high level of plasma lutein and zeaxanthin is associated with a lower risk of AMD [41]. The dietary intake of lutein is associated with a lower risk of different forms of AMD [42,43]. In patients with diabetes, lutein and zeaxanthin supplementation improves retinal structure and function [44,45,46]. Macular pigment optical density, which reflects lutein and zeaxanthin levels in the eye, is lower in patients developing DR [47,48]. However, in ROP, current clinical investigations with small numbers of patients and possibly insufficient doses of lutein did not show any reduction in ROP incidence [49,50,51,52].

In ischemic mouse retinas, lutein supplementation protects neurons from oxidative stress and inflammation [53,54]. In diabetic animals, lutein and zeaxanthin administration reduces oxidative stress and pro-inflammatory markers [55,56,57]. In oxygen-induced retinopathy mice modeling proliferative ROP, lutein supplementation improves retinal revascularization during the hypoxic phase, possibly by affecting the astrocytic template and endothelial tip cell formation [58]. However, no significant change is found in neovascularization [58]. Further exploration regarding the optimal dose, intervention period, and interaction with other supplements is needed to better translate lutein and zeaxanthin supplementation into clinical use.

### 2.3. Others

#### 2.3.1. Resveratrol

Resveratrol, found in red wine, has anti-oxidant properties [59] and protects retinal neurons under oxidative stress. Despite its promising anti-oxidant effects, García-Layana et al. found that in subjects with unilateral wet AMD, after treatment with the original AREDS formulation at concentrations approved in European countries and then further supplemented with resveratrol, DHA, lutein, zeaxanthin, and hydroxytyrosol, there was no beneficial effect on visual acuity compared with the effects of the original European AREDS formulation [60]. Another comparison study on the incidence of CNV between resveratrol- and placebo-administered subjects has been conducted, and study results are pending (NCT02625376).

In preclinical studies, Feng et al. showed that resveratrol treatment can ameliorate retinal ischemia/reperfusion (I/R) injury by modulating the NOD-, LRR-, and pyrin domain-containing protein 3 (NLRP3) inflammasome and Keap1/Nrf2/Ho-1, which regulates cytoprotective responses to oxidative stress [61]. Xie et al. similarly suggested that resveratrol treatment alleviates retinal I/R injury by inhibiting the NLRP3/Gasdermin D/Caspase-1/Interleukin-1β pyroptotic pathway [62].

Resveratrol can reduce vascular dysfunction induced by retinal I/R injury [63]. Lee et al. found that the oral administration of resveratrol inhibits the development of CNV in mice [64]. Resveratrol might inhibit hypoxia-inducible factor-1 alpha (HIF-1α) and vascular endothelial growth factor (VEGF) by blocking the signaling pathway PI3K/Akt/mTOR and by promoting the degradation of HIF-1α protein. HIFs are potential therapeutic molecular targets for ocular neovascularization [65]. HIFs (especially, HIF-1) regulate the expression of many pro-angiogenic genes including VEGF [66]. Further investigations of the inhibitory effects of resveratrol on HIF are needed.

Nagai et al. showed that resveratrol prevents CNV formation by modulating AMP-activated protein kinase (a central regulator of energy homeostasis) [67] in macrophages and other inflammatory cells [68]. We previously found that the oral administration of resveratrol protects against the development of retinal neovascular lesions in very low-density lipoprotein receptor mutant (Vldlr^−/−^) mice [69]. The Vldlr^−/−^ mouse model aspects of macular telangiectasia (MacTel) and neovascular AMD including subretinal neovascularization and photoreceptor degeneration [70,71]. Resveratrol’s protective effects may be through the inhibition of VEGF expression and the suppression of activated retinal endothelial cells.

In DR, Soufi et al. found that the oral administration of resveratrol regulates hyperglycemia, body weight, oxidative stress, and superoxide dismutase activity in the blood and retina of diabetic rats [72]. Furthermore, resveratrol administration prevents the loss of retinal cells determining retinal thickness. Chen et al. demonstrated that resveratrol attenuates retinal pathologic inflammation and ocular damage in diabetes [73]. Jiang et al. reported that resveratrol can reverse the high-glucose-induced inflammatory metabolic memory of human retinal vascular endothelial cells through the activation of the SIRT1/AMPK/NF-κB pathway [74]. Taken together, resveratrol may potentially ameliorate retinal ischemic diseases. Clinical trials are needed to clearly determine the translational value of the preclinical studies.

#### 2.3.2. Vitamin B3 and Nicotinamide Mononucleotide

Vitamin B3 (also known as nicotinamide or niacinamide) is a water-soluble derivative of niacin which is found naturally in many foods. Niacin deficiency is rare in humans. However, the dose required to prevent disease progression might be higher and might vary depending on the disease condition.

A randomized, placebo-controlled, multi-center phase III trial (Nicotinamide in Glaucoma (NAMinG)) will examine the therapeutic effects of nicotinamide on early to moderate open-angle glaucoma progression, evaluating changes in visual fields (NCT05405868). This will be completed in 2026.

In preclinical studies, Williams et al. demonstrate that vitamin B3 administration can improve mitochondrial function to prevent glaucoma in aged mice [75]. Tribble et al. found that nicotinamide improves the metabolic profile in animals with glaucoma by modulating oxidative phosphorylation and mitochondrial size and motility [76]. Ji et al. found that nicotinamide treatment attenuates retinal ischemic damage and light-induced damage to neurons [77]. Saini et al. demonstrated that nicotinamide can ameliorate several disease-related phenotypes in the human iPSC modeling aspects of AMD [78]. Vitamin B3 reduces the production of drusen-related proteins and inflammatory and complement factors and inhibits the increased expression of nucleosome, ribosome, and chromatin-modifying genes.

Nicotinamide can be converted into nicotinamide mononucleotide (NMN) through the rate-limiting enzyme nicotinamide phosphoribosyltransferase (NAMPT) [79]. NMN has been reported to have anti-aging properties [80]. Various clinical trials have been actively conducted with NMN (NCT03151239, NCT04571008, NCT04685096, NCT04862338, and NCT04664361). NMN may also help prevent many experimental eye diseases, such as ischemic retinopathy, corneal injury, glaucoma, and wet and dry AMD [81]. We found that NMN improves retinal function (electroretinography: ERG) in retinal I/R injury and in unilateral common carotid artery occlusion [82,83]. Inflammatory cell recruitment (possibly microglia and/or macrophages) or pathological retinal reactive gliosis under each ischemic disease condition improves with NMN treatment. In a mouse model of unilateral common carotid artery occlusion, retinal nicotinamide adenine dinucleotide (NAD^+^) levels and nuclear factor erythroid 2-related factor 2 (NRF2) expression levels are boosted by NMN treatment. NAD^+^ is a coenzyme for redox reactions that play pivotal roles in cellular metabolism [84]. NRF2 is a strong master transcription factor upregulating anti-oxidant response element-mediated expression of anti-oxidant enzymes [85]. The anti-oxidant effects of NMN might protect against retinal dysfunction in unilateral common carotid artery occlusion-induced retinopathy. Taken together, NMN and vitamin B3 are candidates to suppress retinal ischemic damage. Furthermore, as NMN has reported systemic anti-aging effects in many tissues [86,87,88,89], it is likely to be safe. Clinical studies on retinal ischemic diseases are required.

#### 2.3.3. Vitamin B6

Vitamin B6 (pyridoxine) is a water-soluble vitamin found in many foods (such as peanuts, bananas, salmon, turkey, chicken, and soybeans). In randomized clinical trial data from a large cohort of women at a high risk of cardiovascular disease, daily supplementation with folic acid, pyridoxine, and cyanocobalamin (vitamin B12) reduces the risk of AMD [90]. Another recent study from Ruan et al. showed that vitamin B6 consumption is associated with a reduced risk of DR. In subjects with DR, a higher intake of vitamin B6 is associated with a lower risk of death from all causes and in particular with a lower risk of cardiovascular disease-associated death [91]. Horikawa et al. reported data from the Japan Diabetes Complications Study (JDCS), which found that high vitamin B6 intake is associated with a lower incidence of DR in Japanese patients with type 2 diabetes [92]. This suggests promising protective effects of vitamin B6 supplementation in humans with metabolic diseases.

In a preclinical study, Ibuki et al. demonstrated that long-term vitamin B6 supplementation prevents laser-induced CNV formation in adult mice [93]. Furthermore, vitamin B6 inhibits HIF activation in ocular cells. Vitamin B6-enriched rice bran also showed preventive effects on laser-induced CNV formation in adult mice. Matsubara et al. reported the vitamin B6-mediated suppression of colon tumorigenesis, cell proliferation, and angiogenesis [94]. However, the administration of vitamin B6 decreases retinal function in a murine model of light-induced retinopathy analyzed with ERG [93]. This implies that the dose and duration of administration need further investigation for specific disease conditions.

#### 2.3.4. Vitamin C

Vitamin C (ascorbic acid) is a well-known anti-oxidant nutrient. Vitamin C is vital to the formation of blood vessels, cartilage, muscle, and collagen in bone [95] and is required for the direct and indirect synthesis and metabolism of folic acid, tyrosine, tryptophan, glycine, proline, lysine, carnitine, and catecholamine.

In patients with proliferative diabetic retinopathy (PDR), Park et al. demonstrated that vitreous vitamin C levels are decreased 10-fold. Decreases in vitamin C levels correlate with the severity of macular ischemia [96]. Qian et al. found that central retinal and choroidal thickness is reduced in patients with a vitamin C deficiency [97].

In preclinical studies, Choi et al. found that ascorbic acid is neuroprotective in a rat model of ischemic retinal injury induced by high intraocular pressure [98]. The protective effects may be related to the modulation of neuronal nitric oxide synthase (nNOS) expression in the ischemic retina. Although indirectly related to retinal ischemic diseases, vitamin C may protect against retinal ganglion cell damage through secreted phosphoprotein 1 in glaucoma and in optic nerve damage models [99]. More studies are required to unravel the therapeutic potential of vitamin C supplementation in retinal ischemia.

#### 2.3.5. Lactoferrin

Lactoferrin is a nutrient mainly found in mammalian milk. It is reported to have systemic anti-inflammation, anti-oxidant, and anti-tumor properties in humans [100]. The effects of lactoferrin on the eye need further investigation. Ibuki et al. found that lactoferrin treatment suppresses laser-induced CNV formation in adult mice by modulating HIF-1α [101]. Montezuma et al. demonstrated that endogenous lactoferrin reduces CNV volume in mice, and treatment with exogenous lactoferrin reduces CNV volume in lactoferrin knockout mice [102]. Although indirectly related to retinal ischemic diseases, myopia, nearsightedness, is suppressed by digested lactoferrin or holo-lactoferrin treatment [103,104]. The suppressive effects of latoferrin on myopia progression may be related to anti-inflammation in the eye. Pathologic myopia is a degenerative condition in the sclera, choroid, and RPE caused by the dramatic elongation of axial length in the eye [105], which is associated with a reduction in ocular blood flow [106,107,108]. Although clinical studies are required for targeting ocular ischemic diseases, lactoferrin supplementation may prevent the development and progression of neovascular AMD or other types of macular degeneration.

#### 2.3.6. Crocetin

Crocetin is a natural apocarotenoid dicarboxylic acid found in Gardenia jasminoides fruit and in the crocus flower together with its glycoside, crocin. Crocetin and crocin may prevent and/or treat many metabolic diseases through their strong anti-oxidant effects [109,110]. In a randomized, triple-blind, placebo-controlled primary open-angle glaucoma clinical trial, Mahdiani et al. found that crocin supplementation inhibits disease progression [111]. In preclinical studies, Ishizuka et al. demonstrated that crocetin treatment decreases the number of TUNEL-positive cells and 8-OHdG-positive cells, and the phosphorylation levels of p38, JNK, NF-κB, and c-Jun in ischemic retina seen after I/R injury [112]. Nitta et al. also showed its protective effect in another experimental model of retinal ischemia [113]. In a murine retinal vein occlusion model, the oral and ocular administration of crocetin improved retinal edema by modulating the levels of MMP-9 and TNF-α, and by increasing levels of occludin. A recent study from Wang et al. suggested that the intravitreal administration of crocetin reduces CNV volume and suppresses increased levels of HIF-1α/VEGF and other pro-inflammatory cytokines [114].

Taken together, crocetin may be a promising therapeutic supplement not only for retinal neuronal cell death but also for ocular neovascularization. Further clinical studies are needed.

#### 2.3.7. Taurine

Taurine is a widely distributed natural amino sulfonic acid. In a controlled clinical trial, supplementation with taurine showed possible anti-aging properties [115]. Taurine is abundant in the retina [116] possibly provided by the RPE and Müller cells to photoreceptors [117]. Although the specific roles of taurine in the retina are unclear, taurine depletion has been reported in abnormal ocular conditions [118,119,120,121].

Tao et al. showed that taurine protects against N-methyl-N-nitrosourea-induced photoreceptor cell damage [122]. Froger et al. suggested that taurine supplementation increases the density of retinal ganglion cells in DBA/2J mice (a well-known glaucoma experimental model), in rats with vein occlusion (another glaucoma model), and in P23H rats (a model of retinitis pigmentosa with secondary damage to retinal ganglion cells) in contrast to control groups consuming taurine-free vehicle [123]. Supplementation with taurine may directly help surviving retinal ganglion cells under retinal disease conditions. Taurine also affects vasculature [124,125]. However, taurine has not been well studied in ocular ischemic diseases in a clinical setting.

#### 2.3.8. Palmitoylethanolamide and Peroxisome Proliferator-Activated Receptor α Agonists

Palmitoylethanolamide (PEA) is a naturally occurring fatty acid amide first isolated and described as N-(2hydroxyethyl)-palmitamide [126]. PEA is found in many natural foods, including egg yolks and peanuts. PEA acts through the selective activation of the nuclear receptor peroxisome proliferator-activated receptor α (PPARα) [127].

Ye et al. found that PEA treatment, through PPARα activation, reduces neovascularization in the eye and reduces fibrosis and Müller gliosis in experimental models of proliferative retinopathy and neovascular AMD [128]. Rossi et al. summarized the promising effects of PEA on pattern electroretinogram and on quality of life in patients with glaucoma [129]. Paterniti et al. found that treatment with PEA reduces retinal inflammation marker levels (including VEGF, ICAM-1, and nitrotyrosine) in streptozotocin-induced diabetes in rats [130]. PEA supplementation might reduce ocular inflammation and vascular defects in the eye but clinical studies are needed.

Modulating PPARα may improve retinal ischemia in animals and humans [131,132]. Two PPARα agonists (fenofibrate and pemafibrate) have been examined in many experimental models of retinal ischemia. We found that fenofibrate inhibits cytochrome P450 epoxygenase 2C activity to suppress pathological ocular angiogenesis (especially, neovascularization in the retina and the choroid) [27]. Huang et al. used fenofibrate nano-emulsion eye drops to efficiently suppress retinal vascular leakage as well as ocular neovascularization in mice and rats [133]. Qiu et al. also used fenofibrate-loaded biodegradable nanoparticles to treat experimental DR and neovascular AMD [134]. They reported no toxic effects of its use on the retina structurally and functionally.

Selective peroxisome proliferator-activated receptor α modulator pemafibrate’s promising preventive and protective effects have been confirmed in murine models of retinal I/R injury, carotid artery occlusion-induced ischemic retinopathy, streptozotocin-induced DR, and pathological ocular angiogenesis (CNV and retinal neovascularization) with increases in the serum levels of fibroblast growth factor 21 (FGF21), an important metabolic regulatory hormone [135], and decreases in inflammatory responses [136,137,138,139,140]. In the oxygen-induced retinopathy and retinal I/R injury models, pemafibrate treatment reduces pathologic HIF expression in the eye. This implies that HIF-mediated inflammation and vascular damage are modulated by systemic PPARα activation. In the carotid artery occlusion-induced ischemic retinopathy model, retinal gliosis is modulated by pemafibrate administration. As FGF21 has therapeutic effects on retinal glial remodeling to preserve retinal function in retinal degeneration [141], pemafibrate-induced circulating FGF21 might contribute to glial modulation in carotid artery occlusion-induced ischemic retinopathy. In the streptozotocin-induced DR, synaptophysin expression, one of the integral membrane proteins localized to synaptic vesicles to exert important roles in neurotransmitter delivery [142,143], is preserved by pemafibrate treatment. In the laser-induced CNV model, microglial activation in the choroid and RPE is reduced by pemafibrate administration. Taken together, nutrient supplements to boost PPARα could help prevent retinal ischemic diseases.

#### 2.3.9. Metabolites

Many essential metabolites, including amino acids, hormones, flavonoids, glucose, and vitamins, are made through biotransformation by tissues and by gut microorganisms after food digestion. Harder et al. showed that intraocular pressure can disrupt pyruvate levels in the retina, and that mTOR can be activated during neurodegeneration in the eye [144]. Therefore, oral supplementation with pyruvate or rapamycin (a well-known mTOR inhibitor) may be used to protect against pathological retinal degeneration. Neu et al. found that the dipeptide arginyl-glutamine inhibits retinal neovascularization in oxygen-induced retinopathy. This promising effect is linked with a reduction in retinal VEGF levels [145]. As many metabolic risk factors are involved in the development and progression of ischemic retinopathies [146,147], controlling those factors with food consumption or medications could be an important strategy to prevent retinal ischemic diseases.

## 3. Conclusions

Anti-VEGF drugs are used as first-line treatment for retinal ischemic diseases [148]. Although these drugs are effective, the use of anti-VEGF drugs has several disadvantages, such as transient effects and the need for repeated injections resulting in retinal detachment (in some cases) or endophthalmitis (in rare cases) [149]. As VEGF is a physiologically important protein necessary for the survival of many retinal neurons and Müller glia [150,151,152,153], long-term intravitreal anti-VEGF therapies could be toxic to many cells. Anti-VEGF therapies also have local and systemic adverse effects and in some cases fail to suppress neovascularization or edema [154,155]. Anti-VEGF drugs only work to suppress late-stage ocular neovascularization [156]. The prevention of retinal ischemic diseases needs a different strategy. There is a need to develop therapeutics to prevent ischemic ocular damage.

Nutritional supplements might positively affect pathways involved in inflammation, oxidative stress, neurodegeneration, and even pathologic angiogenesis [157,158]. Many supplements improve biological functions relevant to the prevention and treatment of retinal ischemic damage.

More studies are needed to examine the effects of long-term nutritional supplements on biological functions. Investigations are also necessary on whether and how nutritional supplements reach and directly affect the retina. Although the adverse effects of each supplement must be further examined, many supplements have the potential to positively affect retinal ischemia and systemic metabolic changes [147,159,160,161]. Balanced nutritional supplements may improve retinal function and structure.

The outcomes of nutritional supplementation in experimental studies might be affected by the other components of diet, endogenous hormones, or the fed/fasted status. Tuck et al. emphasized that the nutritional profile of diets affects experimental reproducibility in rodent microbiome experimental research [162]. Even different feeding routines, possibly affecting systemic metabolic hormones, could impact mouse physiology [163]. Also, organ-to-organ communication may be important in modulating biological functions [164].

**Figure 2:**
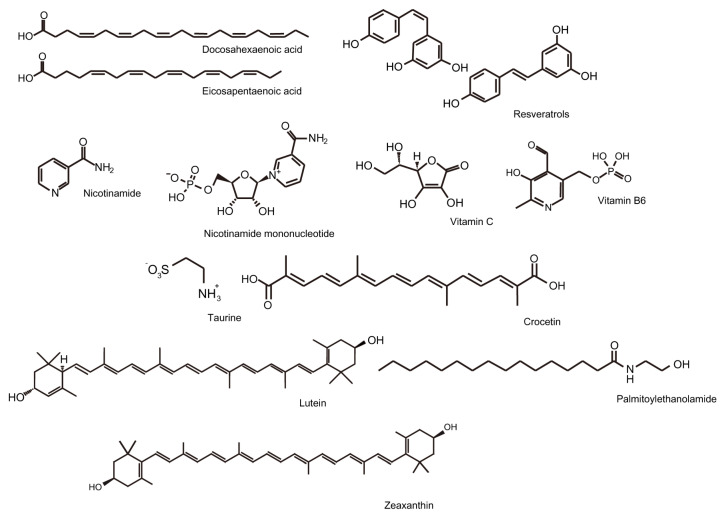
Chemical structures of the nutrients mainly discussed in the current review article. Omega-3 (eicosapentaenoic acid: PubChem 446284, and docosahexaenoic acid: PubChem 445580), lutein (PubChem 5281243), zeaxanthin (PubChem 5280899), resveratrol (PubChem 445154 and 1548910), nicotinamide (PubChem 936), nicotinamide mononucleotide (PubChem 14180), vitamin C or ascorbic acid (PubChem 54670067), vitamin B6 or pyridoxal phosphate (PubChem 1051), taurine (PubChem 1123), crocetin (PubChem 5281232), and palmitoylethanolamide (PubChem 4671) are listed [165,166].

In conclusion, we briefly summarized the therapeutic effects of nutritional supplementation in retinal ischemic diseases with recent experimental evidence (Figure 2). Our review should engender the consideration of nutritional supplementation in the prevention or treatment of retinal ischemic diseases to further develop and conduct in-depth clinical studies.
